# Transcriptomic analysis of brain tissues identifies a role for CCAAT enhancer binding protein β in HIV-associated neurocognitive disorder

**DOI:** 10.1186/s12974-020-01781-w

**Published:** 2020-04-11

**Authors:** Saranya Canchi, Mary K. Swinton, Robert A. Rissman, Jerel Adam Fields

**Affiliations:** 1 Veterans Affairs San Diego Healthcare System, San Diego, CA USA; 2 Department of Neurosciences, University of California San Diego, San Diego, La Jolla, CA USA; 3 Department of Psychiatry, School of Medicine, University of California San Diego, 9500 Gilman Dr., BSB 3009, San Diego, La Jolla, CA 92093-0603 USA

**Keywords:** HIV, Brain, C/EBPβ, Neuron, Astroglia, Neuroinflammation

## Abstract

**Background:**

HIV-associated neurocognitive disorders (HAND) persist in the era of combined antiretroviral therapy (ART) despite reductions in viral load (VL) and overall disease severity. The mechanisms underlying HAND in the ART era are not well understood but are likely multifactorial, involving alterations in common pathways such as inflammation, autophagy, neurogenesis, and mitochondrial function. Newly developed omics approaches hold potential to identify mechanisms driving neuropathogenesis of HIV in the ART era.

**Methods:**

In this study, using 33 postmortem frontal cortex (FC) tissues, neuropathological, molecular, and biochemical analyses were used to determine cellular localization and validate expression levels of the prolific transcription factor (TF), CCAAT enhancer binding protein (C/EBP) β, in brain tissues from HIV+ cognitively normal and HAND cases. RNA sequencing (seq) and transcriptomic analyses were performed on FC tissues including 24 specimens from well-characterized people with HIV that had undergone neurocognitive assessments. In vitro models for brain cells were used to investigate the role of C/EBPβ in mediating gene expression.

**Results:**

The most robust signal for TF dysregulation was observed in cases diagnosed with minor neurocognitive disorder (MND) compared to cognitive normal (CN) cases. Of particular interest, due to its role in inflammation, autophagy and neurogenesis, C/EBPβ was significantly upregulated in MND compared to CN brains. C/EBPβ was increased at the protein level in HAND brains. C/EBPβ levels were significantly reduced in neurons and increased in astroglia in HAND brains compared to CN. Transfection of human astroglial cells with a plasmid expressing C/EBPβ induced expression of multiple targets identified in the transcriptomic analysis of HAND brains, including dynamin-1-like protein (DNM1L) and interleukin-1 receptor-associated kinase 1. Recombinant HIV-Tat reduced and increased C/EBPβ levels in neuronal and astroglial cells, respectively.

**Conclusions:**

These findings are the first to present RNAseq-based transcriptomic analyses of HIV+ brain tissues, providing further evidence of altered neuroinflammation, neurogenesis, mitochondrial function, and autophagy in HAND. Interestingly, these studies confirm a role for CEBPβ in regulating inflammation, metabolism, and autophagy in astroglia. Therapeutic strategies aimed at transcriptional regulation of astroglia or downstream pathways may provide relief to HIV+ patients at risk for HAND and other neurological disorders.

## Introduction

The number of human immunodeficiency virus (HIV) cases has increased to over 34 million individuals worldwide, and neurological disorders remain prevalent despite the advent of combined antiretroviral therapies (ART). While ART has increased the life expectancy of people with HIV (PWH), HIV-associated neurocognitive disorders (HAND) has become more prevalent or remained at the same levels [1, 2]. HAND severity varies from deficiencies that do not affect daily living, asymptomatic neurocognitive impairment (ANI), to more severe neurocognitive diagnoses such as minor neurocognitive disorder (MND), and in rare cases, HIV-associated dementia (HAD) [3]. The identification of novel mechanisms underlying HAND is needed to develop therapeutic strategies for PWH.

Multiple pathogenic mechanisms are implicated as contributing to HAND progression and they may stem from ART-induced neurotoxicity, HIV protein interactions with uninfected bystander cells, low-level viral replication, and neuroinflammation [4, 5]. Untargeted transcriptomic analyses offer the promise of uncovering novel mechanisms that are relevant to HAND in the era of ART. These mechanisms may be missed by more traditional approaches that focus on specific pathways or biomarkers of interest. Characterizing alterations in the transcriptome in brains of HAND cases compared to HIV+ cognitive normal cases could lead to the discovery of important factors or pathways for the development of therapeutic strategies.

Transcription factors (TFs) can contribute to health and disease by regulating the expression genes involved in important pathways. For example, TFs have been implicated in altering function of pathways such as mitochondrial biogenesis, autophagy, and inflammation in multiple neurodegenerative diseases including Alzheimer’s disease, Parkinson’s disease, and HAND. TFs make for promising therapeutic targets because their potential broad effect on gene expression. Moreover, TFs can function in different cell types of the brain. The same TF may affect different pathways in neurons and glia such as neurogenesis and inflammation, respectively. Identification of altered gene expression networks and the TFs involved opens the door for novel techniques to target cell-specific TF expression to restore homeostasis in diseased tissues.

CCAAT enhancer binding protein (C/EBP) β is a prolific TF that is involved in neurogenesis and inflammatory gene expression in the brain [6, 7]. Furthermore, C/EBPβ-mediated gene regulation has been implicated in Alzheimer’s disease, amyotrophic lateral sclerosis, multiple sclerosis, and HAND [6, 8–12]. We previously reported that C/EBPβ expression is increased in brains of HIVE donors, and HIV-relevant stimuli induce C/EBPβ expression in astroglia [11]. We also showed that C/EBPβ contributes to the expression of 60% of a selected panel of interleukin (IL)-1β-induced astroglial inflammatory genes [12]. Other studies have shown that C/EBPβ is regulated in a cell-specific manner to alter neurogenesis, axonal injury, inflammation, and differentiation depending on the cell type [6]. Despite these findings, the specific cell types in which C/EBPβ is functioning in these different neurodegenerative diseases are unknown.

In this study, we extended previous studies to characterize the cellular expression of C/EBPβ in the brains of HAND donors. We observed strong C/EBPβ signal in neurons of control HIV+ brains, but HAND donors presented reduced C/EBPβ signal in neurons, and increased C/EBPβ signal in astroglia. These analyses suggest that HIV-relevant stimuli may have opposite effects on astroglial and neuronal C/EBPβ expression. These alterations in cellular C/EBPβ expression may underlie neurodegeneration and neuroinflammation in HAND patients.

## Methods

### Study population

For the present study, we evaluated brain tissues from a total of 33 HIV+ donors (Table 1) from the National NeuroAIDS Tissue Consortium (NNTC) (Institutional Review Board [IRB] #080323). All studies adhered to the ethical guidelines of the National Institutes of Health and the University of California, San Diego. These cases had neuromedical and neuropsychological examinations within a median of 12 months before death. Subjects were excluded if they had a history of CNS opportunistic infections or non-HIV-related developmental, neurologic, psychiatric, or metabolic conditions that might affect CNS functioning (e.g., loss of consciousness exceeding 30 min and psychosis). HAND diagnoses were determined from a comprehensive neuropsychological test battery administered according to standardized protocols [13].

**Table 1:** Clinical characteristics of a cohort of 33 brain specimens from people with HIV

	Cognitive normal (***n*** = 10)	Asymptomatic neurocognitive impairment (***n*** = 10)	Minor neurocognitive dysfunction (***n*** = 10)	HIV-associated dementia (***n*** = 3)
**Age**	41.7 ± 8.1	41.7 ± 10.8	43.1 ± 6.7	40.7 ± 2.1
**Sex (fm:m)**	0:10	1:09	1:09	1:02
**Postmortem interval**	33.3 ± 67.5	21.4 ± 28.4	15.5 ± 14.4	9.3 ± 3.01
**Education**	13.0 ± 2.3	12.22 ± 3.4	12.0 ± 3.46	16.0 ± 3.5
**CD4**	151.6 ± 151.2	68.7 ± 73.9	35.3 ± 55.4	98.0 ± 155.1
**VL (log)**	3.3 ± 1.6	3.9 ± 1.3	5.0 ± 0.8	4.4 ± 2.4
Duration on ART (months)	53.3 ± 28.2	56.3 ± 86.5	35.7 ± 19.6	16.0 ± 0.0

### Neuromedical and neuropsychological evaluation

Participants underwent a comprehensive neuromedical evaluation that included assessment of medical history, structured medical and neurological examinations, the collection of blood, cerebrospinal fluid (CSF), and urine samples, as previously described [13, 14]. Clinical data (plasma viral load [VL], postmortem interval, CD4 count, global, learning and motor deficit scores [GDS, LDS, and MDS]) were collected for the HAND donor cohorts.

Neuropsychological evaluation was performed, and HAND diagnoses were determined via a comprehensive neuropsychological test battery, which was constructed to maximize sensitivity to neurocognitive deficits associated with HIV infection [see [13] for a list of tests]. Raw test scores were transformed into demographically adjusted T-scores, including adjustments for age, education, gender, and race. These demographically adjusted T-scores were converted to clinical ratings to determine presence and degree of neurocognitive impairment on seven neurocognitive domains, as previously described [13]. As part of the neuropsychological battery, participants also completed self-report questionnaires of everyday functioning (i.e., Lawton and Brody Activities of Daily Living questionnaire [15]; and/or Patient’s Assessment of Own Functioning (PAOFI) [16, 17];). Participant’s performance on the neuropsychological test battery and their responses to the everyday functioning questionnaires were utilized to assign HAND diagnoses following established criteria [18], i.e., HIV-associated asymptomatic neurocognitive impairment (ANI), HIV-associated mild neurocognitive disorder (MND), and HIV-associated dementia (HAD).

### Immunoblot of human brain specimens

Frontal cortex tissues from human brains were homogenized in lysis buffer (1.0 mmol/L HEPES; Gibco, cat. no. 15630-080), 5.0 mmol/L benzamidine, 2.0 mmol/L 2-mercaptoethanol (Gibco, cat. no. 21985), and 3.0 mmol/L EDTA ((Omni pur, cat. no. 4005), 0.5 mmol/L magnesium sulfate, 0.05% sodium azide; final pH 8.8). In brief, as previously described [19], tissues from human brain samples (0.1 g) were homogenized by sonication for 15 s in 0.7 ml of lysis buffer containing phosphatase and protease inhibitor cocktails (Calbiochem, cat. no. 524624 and 539131). Samples were precleared by centrifugation at ×2000*g* for 5 min at room temperature. The supernatant was collected as representing the whole lysate.

After determination of the protein content of all samples by bicinchoninic acid assay (Thermo Fisher Scientific, cat. no. 23225) and denaturation in lamellae sample buffer, samples were loaded (20 μg total protein/lane) on 4–12% Bis-Tris gels (Invitrogen, cat. no. WG1402BX10) and electrophoresed in 5% HEPES running buffer and transferred onto PVDF membrane with iBlot transfer stacks (Invitrogen, cat. no. IB24001) using NuPage transfer buffer (ThermoFisher Scientific, cat. no NP0006). The membranes were blocked in 5% BSA in phosphate-buffered saline-tween 20 (PBST) for 1 h. Membranes were incubated overnight at 4 °C with primary antibody. Following visualization, blots were stripped and probed with a mouse monoclonal antibody against β-actin (ACTB; Sigma-Aldrich, cat. no. A5441) diluted 1:2000 in blocking buffer as a loading control. All blots were then washed in PBST, and then incubated with species-specific IgG conjugated to HRP (American Qualex, cat. no. A102P5) diluted 1:5000 in PBST and visualized with SuperSignal West Femto Maximum Sensitivity Substrate (ThermoFisher Scientific, cat. no. 34096). Images were obtained, and semi-quantitative analysis was performed with the VersaDoc gel imaging system and Quantity One software (Bio-Rad).

### Immunohistochemistry and double immunofluorescence

Free-floating 40-μm thick vibratome sections of human brains were washed with phosphate-buffered saline (PBS) 3 times, pre-treated for 20 min in 3% H_2_O_2_, and blocked with 2.5% horse serum (Vector Laboratories, cat. no. S-2012) for 1 h at room temperature. Sections were incubated at 4 °C overnight with the primary antibody C/EBPβ (Santa Cruz Biotechnology; C-150) diluted in blocking buffer. Sections were then incubated in secondary antibody Immpress HRP Anti-rabbit IgG (Vector, cat. no. MP-7401) for 30 min, followed by peroxidase (HRP) substrate made with DAB peroxidase (HRP) substrate kit as per manufacturer’s instructions (Vector, cat. no. SK-4800). Control experiments consisted of incubation with secondary antibody only. Tissues were mounted on Superfrost plus slides (Fisherbrand, cat. no. 12-550-15) and coverslipped with cytoseal (Richard Allen Scientific, cat. no. 8310-16). Immunostained sections were imaged with a digital Olympus microscope to identify C/EBPβ immunoreactivity.

Double immunolabeling studies were performed as previously described [20] to determine the cellular localization of C/EBPβ. For this purpose, vibratome sections of human brains were immunostained with antibodies against C/EBPβ with GFAP (Cell Signaling Technology; catalog no. 3670), and MAP2 (Santa Cruz Biotechnologies, cat. no. sc-32791). Sections were then reacted with fluorescent secondary antibodies, goat anti mouse IgG 488 (Invitrogen, cat. no. A11011), and goat anti rabbit IgG 568 (Invitrogen, cat. no. A11036). Sections were mounted on superfrost plus slides and coverslipped with vectasheild (Vector, cat. no. 1000). Sections were imaged with a Zeiss 63× (N.A. 1.4) objective on an Axiovert 35 microscope (Zeiss) with an attached MRC1024 laser scanning confocal microscope system (BioRad, Hercules, CA). An examiner blinded to sample identification analyzed all immunostaining. Ten fields of view were analyzed, and a minimum of 20 cells (astrocytes and neurons) per slide were examined to analyze colocalization. The percent colocalization was determined using the Image J software with the SQASSH plug-in, as previously described. Double immunolabeling of C/EBPβ with the microglial marker IBA1 was excluded due to technical difficulties and high level of non-specific signal when using the two antibodies simultaneously. Future investigation will focus on identifying levels of C/EBPβ in microglial cells.

### In vitro studies of human astrocytes and neuronal cells

In vitro models for human astrocytes generated from fetal tissue are important for controlling for differences in genetic background, which cannot be controlled for by using cell lines. Moreover, astrocytes from human tissue are directly relatable to human disease, unlike astrocytes generated from rodents. The cell model for astrocytes was approved by the University of California San Diego Human Research Protections Program and the National Institutes of Health as part of a grant actively funded by the National Institute for Mental Health. Astrocytes were isolated from fetal human brain tissue from elective terminated pregnancy between 12 and 16 weeks of gestation, acquired from Advanced Bioscience Resources. Donors gave written informed consent for research use of the cells and tissue. Tissue was fragmented and mechanically dissociated using a scalpel and washed 3 times with HBSS holding media (Gibco, cat. no.14175-095) with 1 mM Glutamax (Gibco, cat. no. 35050-061), 20 μg/mL gentamicin (Gibco, cat. no. 15710-064), and 5 mM HEPES (Gibco, cat. no. 15630-080). The tissue was homogenized with the addition of 15 mL of 0.25% trypsin EDTA (Gibco, cat. no. 25200-056) for 5 min in a 37 °C incubator. After 5 min, 1 mL of a trypsin inhibitor (Roche, cat. no. 10109) and 24 mL of DMEM media (Gibco, cat. no. 11960-044) with human serum (Corning, cat. no. 35-060-cl) were added. The mixture was then centrifuged for 5 min at 4 °C to pellet the cells. Supernatant was removed and discarded, and the cells were resuspended in 5 ml of DMEM media and strained with a 70 μM strainer (Falcon, cat. no. 352350). The cell suspension was underlaid with 7 ml of a solution of filtered 8% BSA in PBS, and cells were centrifuged at 1 × 10^4^ rpm at 4 °C for 10 min. The supernatant was removed, and the cells were resuspended in DMEM media with human serum. Astrocytes were plated at a density of 1 × 10^7^/T75 flask and cultured as adherent monolayers. After 1 week, the astroglia DMEM media with human serum was replaced with DMEM media with 10% fetal bovine serum (FBS) (Gibco, cat. no. 16000044) and 1% penicillin/streptomycin (P/S) (Corning, cat. no. 30-001-CI-1). Every 3 days, a half media exchange was performed on each cell type. Astrocytes were routinely tested purity and consistently found to be > 95% pure by immunostaining for GFAP.

As previously described [21], B103 cells (rat neuroblastoma) were cultured at 37 and 5% CO2. B103 rat neuroblastoma cells were used here for the cholinergic and GABAergic phenotypes [22], both of which are implicated in frontal cortex and basal ganglia function [23, 24] and relevant to HAND [25, 26]. B103 cultures were grown in DMEM with 5% FBS.

### Transfection of astroglia with pC/EBPβ

Astrocytes were split into 12 well plates at 500,000 cells/well on the day prior to transfection. Astrocytes were transfected using Lipofectamine 3000 (Thermo Fischer Scientific, cat. no. L3000075). Lipofectamine 3000 and an empty lentiviral expression plasmid (p) as a control or pC/EBPβ (OriGene Technologies, Rockville, MD; CAT. SC319561) (1 μg) with p3000 were diluted separately in Opti-Mem Media and then mixed together at a 1:1 ratio and left to incubate for 15 min at room temperature. After 15 min, the plasmid-lipid complexes were added to the cells. Three days after transfection, RNA was isolated from astroglia. Genes were selected for validation by real-time polymerase chain reaction (rt^2^PCR) based on bioinformatic analyses of transcriptome of the PWH indicating they were potential targets of C/EBPβ transcription in astrocytes. Moreover, all transcripts tested are implicated in HAND, neuroinflammation, or both.

### RNA isolation and real-time reverse transcription polymerase chain reaction

Astroglia were split into 12 well plates at 5 × 10^5^ cells/well for RNA isolation. Three days after transfection, media was removed, and the cells were washed once with PBS. RNA was extracted with RNeasy plus mini kit (Qiagen, cat. no. 74136) according to manufacturer’s instructions and analyzed for purity and concentration with a spectrophotometer. RNA was reverse transcribed into cDNA with a high capacity cDNA Reverse Transcription Kit (Life technologies, cat. no. 4358813) as per manufacturer’s instructions. Taqman gene expression assays were performed using the StepOnePlus sequence-detection system (Life Technologies), using primers specific to DNM1L (Taqman, cat. no. hs00174131), IRAK1 (Taqman, cat. no. hs001155570), BCL11B (Taqman, cat. no. hs01102259), PINK1 (Taqman, cat. no. hs00260868), and ActB (Applied Biosystems, cat. no.1612290). A master mix was made using 5 μl of 2× fast advanced master mix (Thermofisher Scientific, cat. no. 4444557), 0.5 μl of 20× primers, and 2 μl of water per reaction well. To each well of a microamp fast optical plate (Applied Biosystems, cat. no. 4346907), 8 μl of master mix and 2 μl of cDNA were added (the reactions were carried out at 48 °C for 30 min and 95 °C for 10 min, followed by 40 cycles of 95 °C for 15 s and 60 °C for 1 min). Samples were analyzed in duplicate. Fold changes were calculated using the comparative CT method.

### Exposure of astrocytes and neurons to HIV recombinant proteins

Cells (astrocytes or B103 neuronal cells) were plated on 12 well plates at 800,000 cells per well and treated for 24 or 96 h with vehicle or recombinant gp120 (100 ng/mL; clade E, cat. no. 2968), nef (100 ng/ml; cat. no. 11478), or Tat (10 ng/ml; cat. no. 2222). Total RNA and cytosolic and nuclear enriched lysate fractions were isolated as described.

### Immunoblot of cell lysates

Recombinant proteins were acquired from the NIH AIDS Reagents program. Following treatment with recombinant proteins or transfection with plasmids, cells were washed with sterile PBS and detached using 0.25% trypsin EDTA (Gibco, cat. no. 25200-056). Cells were collected using DMEM media with 10% fetal bovine serum (FBS) (Gibco, cat. no. 16000044) and 1% penicillin/streptomycin (P/S) (Corning, cat. no. 30-001-CI-1). Cells were then centrifuged at 10,000 rpm for 30 s. The supernatant was discarded, and cells were washed with 700 μl of PBS followed by centrifugation at 10,000 rpm for 30 s. The PBS was removed, and cells were lysed using a solution of 0.1% Triton-X in PBS with the addition of protease inhibitors. Cells were then centrifuged again at 10,000 rpm for 30 s. The supernatant was retained as the cytosolic-enriched fraction. The pellet was resuspended in lysis buffer, sonicated, and retained as the nuclear-enriched fraction. After protein concentration was determined using bicinchoninic acid assay (Thermo Fisher Scientific, cat. no. 23225) and samples were denatured in lamellae sample buffer (Bio Rad, cat. no. 1610747), cytosolic and nuclear fractions were loaded (10 μg total protein/lane) on 4–15% Criterion TGX stain free gels (Bio Rad, cat. no. 5678085) and electrophoresed in Tris/Glycine/SDS running buffer (Bio Rad, cat. no. 161-0772) and transferred onto PVDF membrane with Bio Rad transfer stacks and transfer buffer (Bio Rad, cat. no 1704275) using Bio Rad Trans Blot Turbo transfer system. After the transfer, total protein was imaged using Bio Rad ChemiDoc imager under the stain free blot setting for normalization purposes. The membranes were then blocked in 1% casein in tris-buffered saline (TBS) (Bio Rad, cat. no. 1610782) for 1 h. Membranes were incubated overnight at 4 °C with primary antibodies, C/EBPβ, DNM1L, and ACTB, diluted in blocking buffer. All blots were then washed in PBST and then incubated with species-specific IgG conjugated to HRP (American Qualex, cat. no. A102P5) diluted 1:5000 in PBST and visualized with SuperSignal West Femto Maximum Sensitivity Substrate (ThermoFisher Scientific, cat. no. 34096).

### Antibodies

The following antibodies were used in immunoblot, immunohistochemistry, or both: C/EBPβ (Santa Cruz Biotechnology Inc. [SCBT]; catalog no. sc-7962/no. sc150), GFAP (Cell Signaling Technology; catalog no. 3670), MAP2 (SCBT, catalog no. sc-32791), DNM1L (SCBT; catalog no. sc-32898), and β-actin (ACTB; Sigma-Aldrich; catalog no. A2228).

### Statistical analysis

All the analyses of images were conducted on coded samples blinded to the examiner. After the results were obtained, the code was broken, and data were analyzed with the Prism software. Comparisons among groups were performed with one-way ANOVA with post-hoc Fisher test and unpaired Student’s *t* test where appropriate. All results were expressed as mean ± SEM. The differences were considered to be significant if *p* values were < 0.05.

### Gene expression analysis

Total RNA was isolated from 50 mg of postmortem brain tissues from Broddmann Area 46 using the Qiagen RNeasy Lipid Tissue Kit per manufacturer’s instructions (Qiagen; cat no. 74804). The mRNA libraries were generated by the UC San Diego Institute for Genomic Medicine. RNA-seq data (75 bp single end reads with coverage of 20 million) was obtained from RNA extracted from the frontal cortex of MND and HIV+ cognitive normal subjects (CNHIV+). The quality of the raw FASTQ files was assessed using FASTQC v0.11.8. Adapters and low-quality reads were trimmed using a kmer approach as implemented in BBDuk v38.62. Transcripts were quantified using quasi-mapping mode of Salmon v0.14.1 [27] and summarized to gene counts for downstream analysis using the *tximport* v1.10.0 [28] package. Genes were retained in the analysis if they achieved counts per million (cpm) > 1 in at least half of the brain samples. Effective library sizes were estimated by TMM scale normalization prior to analysis to estimate observational weights [29, 30]. Surrogate variables representing latent noise were estimated using *sva* v package [31]. For the log-transformed expression data with precision weights, per-gene linear regression models were fit to account for the effects of cognitive impairment status after adjustment for unmodeled variation sources. Test for statistical significance was achieved by implementation of a Bayesian strategy of Lönnstedt and Speed as implemented in *R* package *limma* v3.38.3 [32]. Significance was defined by using an adjusted *p* value cut-off of 0.05 after multiple testing correction using a moderated t-static in limma.

To identify the underlying biological functions enriched in frontal cortex of MND relative to cognitive normal, Gene Set Enrichment Analysis (GSEA) was implemented which identifies the enrichment of functionally defined gene sets using a modified Kolmogorov-Smirnov statistic [33] and the Molecular Signature Database (MSigDb v6.0). Statistical significance after adjusting for multiple testing is defined at FDR < 0.05. Gene set-based permutation test of 1000 permutations was applied. Hypergeometric test was utilized to test the statistical significance of the enriched biological process and pathways identified for the unique differential expressed genes for each group [34]. Overrepresentation enrichment analysis was conducted using the full set of detected genes as the reference gene set, corrected for multiple testing using the *Benjamini-Hochberg* procedure, and FDR < 0.05 was considered significant. For the identified transcriptional factors (TF) dysregulated in MND, differentially regulated targets were obtained using the experimentally validated TF binding profiles from the ChEA and ENCODE databases [35, 36]. A database of gene expression in mature human astrocytes was created [37] with threshold gene expression set at 1 FPKM per sample. The astrocyte marker genes were ranked according to the overall expression across all samples. Astrocyte marker genes were identified from C/EBPβ targets, and hypergeometric test was used to analyze the functional pathway. All analyses were completed on the R statistical software (v3.6.1) [38].

## Results

### Clinical and neuropathological characteristics of HIV+ donors

A total of 33 HIV+ autopsy cases were obtained through the NNTC and analyzed in order to assess relevant differences between postmortem brain samples from HIV patients. The brain tissues are characterized by age, sex, postmortem interval, education, VL, and CD4+ cell count (Table 1). The average age of each group did not differ significantly, and most of the cases were male. The postmortem interval (PMI) also did not differ significantly between the groups. However, the average and standard deviation of PMI for the CN group was brought up by one case with a PMI of 200 h. Education level also did not differ significantly between groups. The differences in CD4+ cell count between the groups were robust, with MND having the lowest average (*p* = 0.102 versus CN). Similarly, the differences in VL between groups approached significance with the MND group (*p* = 0.061 versus CN) having the highest average. The differences between groups regarding duration on ART are not significant, although the MND group duration on ART was reduced compared to the CN group. Data were analyzed using one-way ANOVA and Tukey’s test for multiple comparisons [39].

### C/EBPβ levels are increased in astroglia and decreased in neurons in HAND cases

**Fig. 1:**
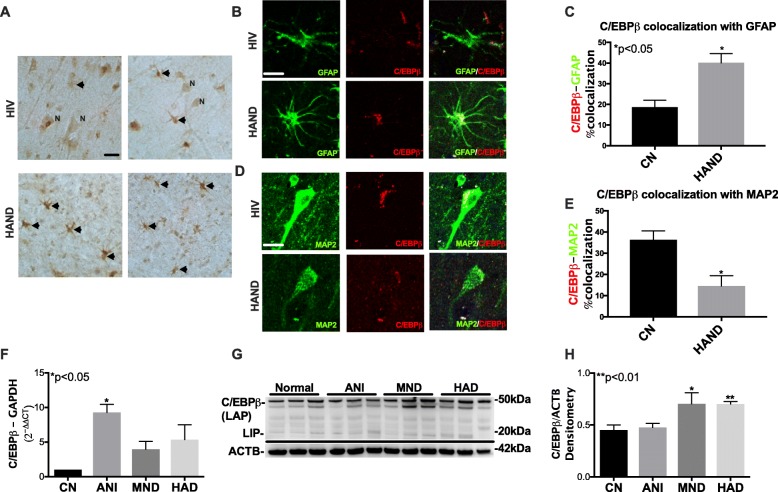
C/EBPβ levels are increased in the astroglia and decreased in neurons in HAND cases. **a** Vibratome sections of frontal cortex tissues from HIV CN and HAND cases immunolabeled for C/EBPβ. **b** Vibratome sections from the frontal cortex from HIV CN and HAND cases double immunolabeled for GFAP (green) and C/EBPβ (red). **c** The percent of C/EBPβ colocalizing with GFAP for CN versus HAND cases. **d** Double immunolabeling was for MAP2 (green) and C/EBPβ. **e** Percent colocalization comparing CN and HAND cases. **f** RNA expression of C/EBPβ normalized to GAPDH plotted by neurocognitive status. **g** Western blot for C/EBPβ and ACTB using lysates of frontal cortex tissues. **h** Band intensities for C/EBPβ plotted by neurocognitive status after normalization to ACTB. Statistical significance was determined by an unpaired *t* test when comparing two groups and by one-way ANOVA when comparing more than two groups (*p < 0.05, **p < 0.01)

To determine the expression patterns of C/EBPβ in the frontal cortex of brains from control HIV+ donors and HAND donors, we performed immunostaining for C/EBPβ in the frontal cortex. C/EBPβ signal was largely localized to pyramidal neurons, with a smaller proportion of the C/EBPβ signal in cells with glial morphology (black arrow), in brains from HIV+ donors with no cognitive impairment compared to HAND cases (Fig. 1a). In contrast, C/EBPβ signal is almost exclusively emanating from cells with glial morphology in brains from HAND donors (Fig. 1a). To determine the cellular expression of C/EBPβ in the brains of HAND patients, we double immunolabeled the tissue sections for C/EBPβ (red) with astroglia (GFAP) or neurons (MAP2). C/EBPβ signal is faint in the nuclei of astroglia (GFAP+ cells) in brains from HIV+ cognitively normal cases, but the C/EBPβ signal in astroglia was robust in the brains from HAND cases (Fig. 1b). Quantification of colocalization of red and green signals showed that approximately 20% of C/EBPβ (red) signal colocalized with GFAP (green) signal in CN brains compared to approximately 40% in HAND brains **(***p* < 0.05**;** Fig. 1c)**.** C/EBPβ signal was strong in neurons (MAP2+) in brains from HIV+ CN cases, but the C/EBPβ signal was less intense in neurons of HAND cases (Fig. 1d). Quantification of C/EBPβ+ neuronal cells revealed a 70% decrease in C/EBPβ colocalization with MAP2 in brains from HAND donors compared to brains from control HIV+ donors, respectively (*p* < 0.05; Fig. 1e).

We have previously reported that C/EBPβ levels are increased in the brains of HIV+ donors compared to control [12]. To confirm previous data and determine C/EBPβ levels in this cohort, we isolated total RNA and protein from the brain tissues and analyzed for C/EBPβ mRNA and protein levels by RT^2^PCR and immunoblot, respectively. C/EBPβ mRNA levels were increased 9-, 4-, and 5-fold in brain tissues from ANI, MND, and HAD donors, respectively (*p* < 0.05; Fig. 1f). C/EBPβ protein detection by immunoblot revealed similar levels of the full-length C/EBPβ isoform in normal and ANI tissues, but the bands corresponding to the full-length isoforms are more intense in the tissues from the MND and HAD brains (Fig. 1g). Quantification of the full-length bands showed similar C/EBPβ levels in normal and ANI tissues, but the intensity of full-length bands was increased by 40% in tissues from MND and HAD brains (*p* < 0.01; Fig. 1h).

### Molecular signature of MND involves coordinated biological response and identifies transcriptional role of C/EBPβ

**Fig. 2:**
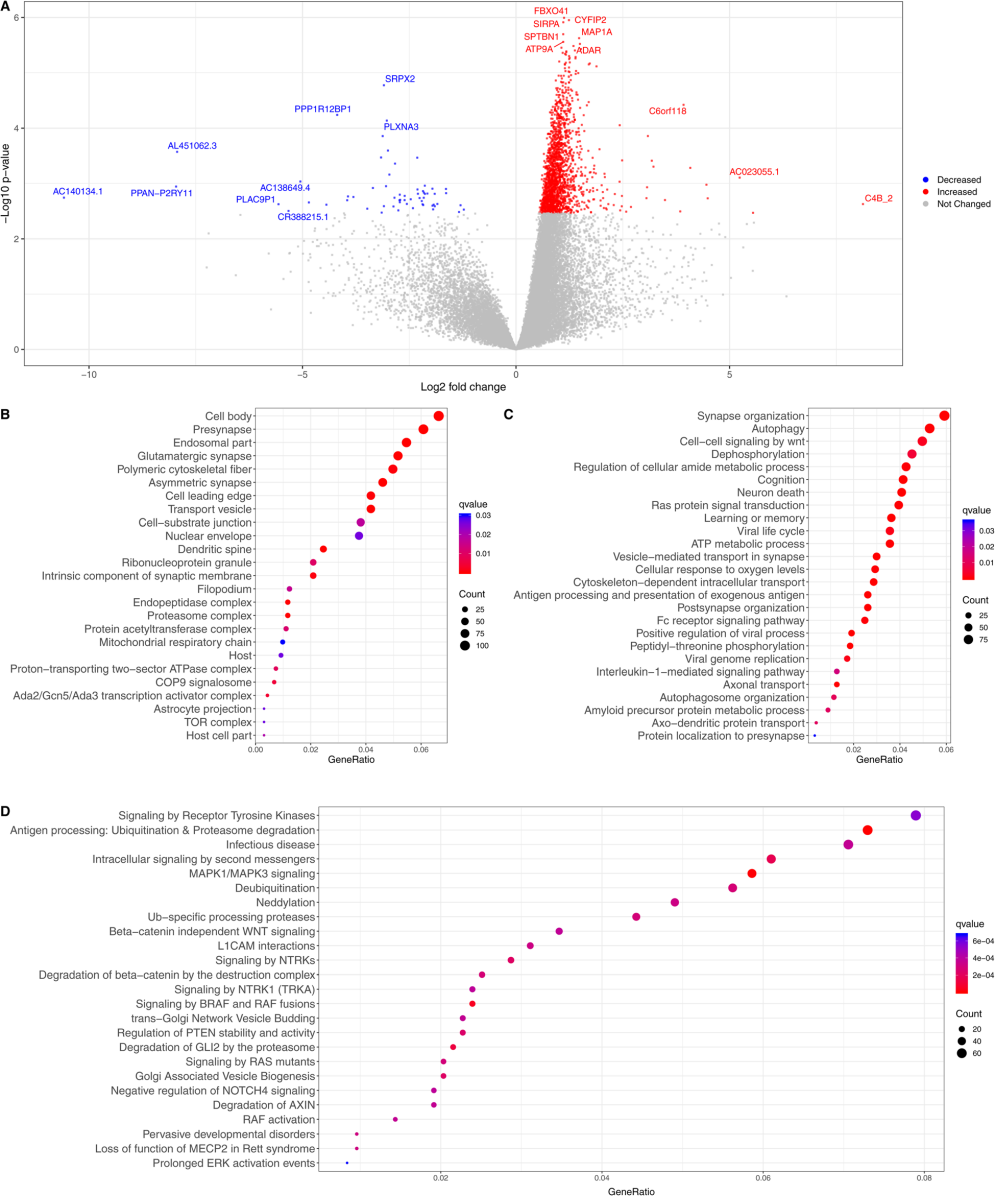
Global transcriptomic changes in HIV-associated minor neurocognitive disorder (MND) relative to controls. **a** Volcano plot of entire set of detected genes where each point represents the difference in expression (fold change) between MND and CN HIV+ subjects plotted against the levels of statistical significance. Upregulated genes are represented in red, downregulated genes in blue, and top genes in each spectrum are highlighted. **b–d** Gene ontology enrichment corresponding to cellular components **(b)** and biological process **(c)** across the differential genes in MND. Biological pathway analysis of differentially regulated C/EBPβ targets based on REACTOME database **(d)**. The terms are arranged by a number of differentially expressed genes associated to an enriched term and *q* values where FDR < 0.05 was considered significant. For full list of gene ontology terms and pathways, see Supplementary Tables S2, S3, S6

After filtering genes with low counts, adjusting for latent variables and multiple testing, a total of 1861 genes were differentially expressed in MND relative to HIV+ cognitively normal subjects (Fig. 2**a,****Table S1**). The top upregulated genes are involved in immune response along with RNA editing which is vital for viral replication while the downregulated genes have role in synaptic maintenance along with novel pseudogenes. Some of the highly upregulated genes including TRIM69, CTSB, B2M, UBE2L6, HLA, and BTB3A3 have been previously reported to be associated with HAND [40]. The subcellular distribution of differentially expressed genes was predominantly sequestered in cell junctions including synapses and associated with organelle membranes like the proteasome, mitochondria, and the lipid-protein complex (Fig. 2**b,****Table S2**). Functional analysis of the gene ontology revealed enrichment in protein processing, synaptic transmission, metabolic, and immune processes (Fig. 2c**,****Table S3**). Viral processes that allow for the survival of the virus including replication of genome and translation of viral mRNA by host ribosomes were also upregulated. Analysis of the disease-gene associations showed the differentially regulated genes were also implicated in other ailments with impaired cognition (**Table S4**). Specifically, Alzheimer’s disease and tauopathy were enriched in the disease ontology (**Table S4**), suggesting potential convergence of the two pathologies [41].

Transcriptional factor (TF) analysis resulted in identification of twenty-six transcriptional regulators whose targets were enriched in MND and who were themselves differentially expressed (Table S5). C/EBPβ, which we have previously shown to be associated with HIVE, was upregulated and had 1308 targets that were differentially expressed in MND. Pathway analysis using REACTOME and KEGG database identified the range of biological perturbations related to targets of C/EBPβ (Fig. 2d, S1**,** Table S6). In addition to regulating the expression of genes involved in immune and inflammatory response, targets of C/EBPβ are broadly involved in metabolism of protein and RNA, cell cycle, response to external stimuli, and intracellular transport. While the genes corresponding to immune functions and autophagy were upregulated, the downregulated gene set correspond to perturbed sphingolipid metabolism and ceramide production, consistent with the observation in neural cells in HAND [42].

**Fig. 3:**
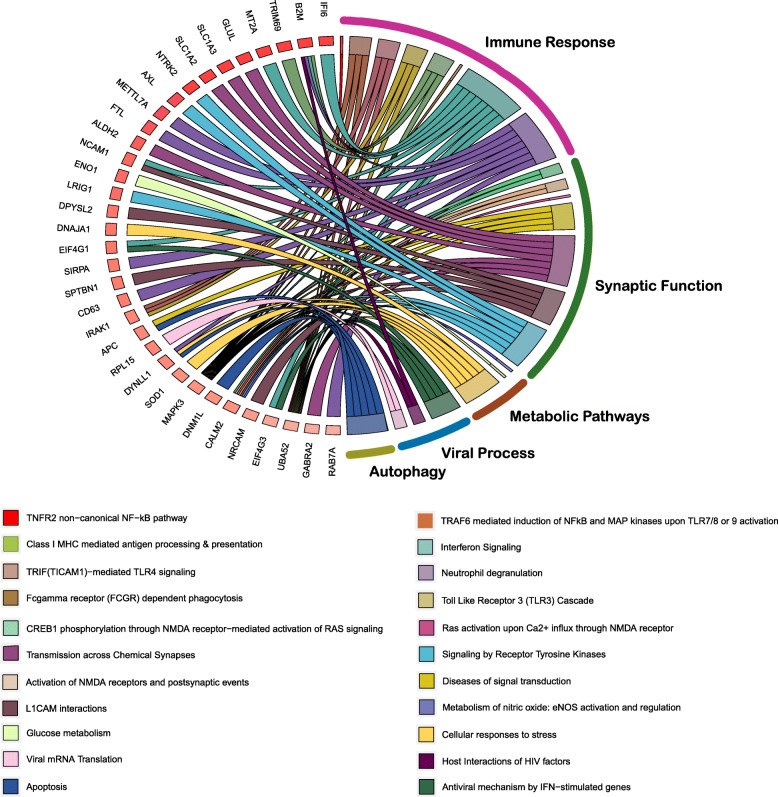
C/EBPβ targets in astrocytes are enriched for immune, metabolic, and signal transduction pathways. Chord diagram showing the enriched REACTOME pathways for differentially regulated C/EBPβ targets that are also astrocyte marker genes. For chord diagrams, individual pathways are shown in the right, and the enriched genes within the pathways are shown on the left. Squares following gene symbols represent the difference in expression between MND and CNHIV+ subjects. Complete list of the astrocyte marker genes that are also C/EBPβ targets is in Supplementary Table S7, and for enriched pathways corresponding to astrocyte marker genes, see Supplementary Table S8

To further identify the role of C/EBPβ in astrocytes based on analysis of the protein expression, astrocyte marker genes from the C/EBPβ targets were identified using a custom database of gene expression in mature human astrocytes [37]. From the targets of C/EBPβ, a total of 1005 genes were astrocyte specific and almost all were upregulated (**Table S7**). In addition to the expected immune response, enriched pathways corresponded to metabolic function, signal transduction, RNA metabolism, and autophagy (Fig. 3**,****Table S8**). Subsequently, we find identified upregulation of KCNQ3, a member of the potassium voltage-gated channel along with glutamate processing machinery including GLUL which converts neurotoxic glutamate to non-toxic glutamine and GRINA which is a subunit of glutamate ionotropic receptor and glutamate transporters (SLC1A2, SLC1A3). Taken together, these results show the comprehensive network of altered downstream effects of C/EBPβ in astrocytes.

### Gene expression is altered in astroglia that over express C/EBPβ

**Fig. 4:**
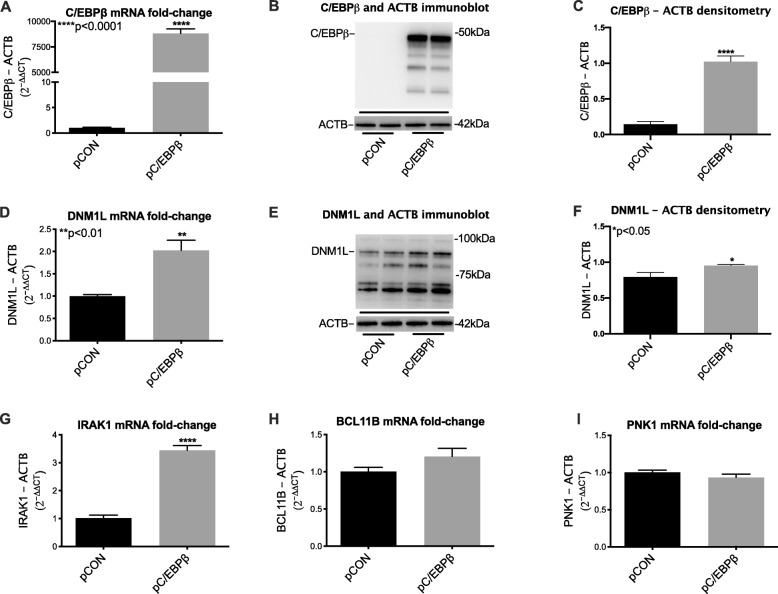
Gene expression is altered in astroglia that overexpress C/EBPβ. **a** Relative expression of mRNA for C/EBPβ in astrocytes transfected with a control plasmid or a plasmid encoding the C/EBPβ gene. **b** Immunoblot for C/EBPβ using whole lysates from control astrocytes and astrocytes overexpressing the C/EBPβ gene. **c** Quantification of densitometry of the ~ 42 kDa band corresponding to C/EBPβ protein. **d** Relative expression of mRNA for DNM1L in astrocytes transfected with a control plasmid or a plasmid encoding the C/EBPβ gene. **e** Immunoblot for DNM1L using whole lysates from control astrocytes and astrocytes overexpressing C/EBPβ gene. **f** Quantification of densitometry of the ~ 90 kDa band corresponding to DNM1L protein. **g**–**i** Relative mRNA expression levels for IRAK1**,** BCL11B, and PINK1 in astroglia transfected with control plasmid of pC/EBPβ. Statistical significance was determined by an unpaired *t* test (*p < 0.05, **p < 0.01, ****p < 0.0001)

After transfecting astroglia with plasmids, control, or expressing C/EBPβ, RNA was extracted and transcribed into cDNA, which was used to measure relative levels of target genes by rt^2^PCR. In parallel experiments, protein was isolated from transfected cells for validation of gene expression at the protein level. C/EBPβ overexpression was confirmed by measuring levels of C/EBPβ relative to ActB (Fig. 4a). Immunoblot of whole lysates from astroglia revealed that transfection with pC/EBPβ-induced robust increase in fold change of C/EBPβ protein (Fig. 4b). Densitometry analyses of the large band corresponding to C/EBPβ revealed a ~ 10-fold change increase compared to cells transfected with control plasmid (Fig. 4c). Overexpression of C/EBPβ also induced a significant increase in fold change of DNM1L mRNA transcripts and protein (Fig. 4d, e). Densitometry analyses of the large band corresponding to C/EBPβ revealed a ~ 20% fold change increase compared to cells transfected with control plasmid (Fig. 4f). Overexpression of C/EBPβ also induced a significant increase in fold change of IRAK1 mRNA compared to control (Fig. 4g). Increases in RNA expression for these markers suggest that increases in C/EBPβ in astroglia may increase immune response and affect mitochondrial dynamics and mitophagy. However, no significant difference was found between pC/EBPβ and control for BCL11B, an immune regulator, and PINK1, a protector against mitochondrial dysfunction (Fig. 4h, i).

### HIV Tat reduces neuronal and increase astroglial C/EBPβ expression in vitro

**Fig. 5:**
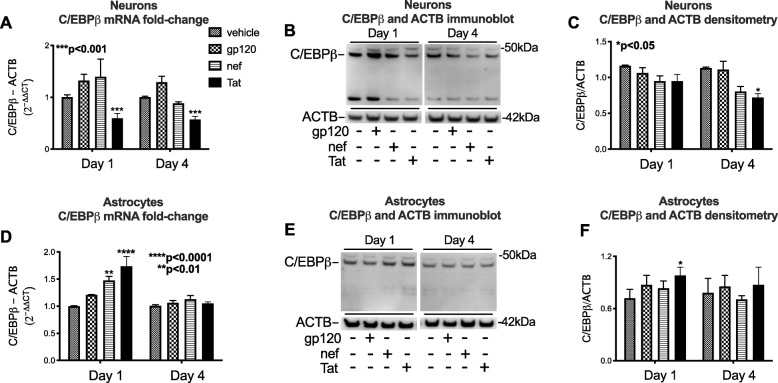
HIV Tat reduces neuronal and increases astroglial C/EBPβ mRNA and protein in vitro. Neuronal and astroglial cells were treated with recombinant HIV proteins (gp120, nef or Tat) for one or four days. **a** Relative expression of mRNA for C/EBPβ in neurons exposed to recombinant HIV proteins compared to vehicle-treated neurons. **b** Immunoblot for C/EBPβ using nuclear-enriched lysates from neurons exposed to vehicle or recombinant HIV proteins. **c** Quantification of densitometry of the the ~ 42 kDa band corresponding to C/EBPβ protein. **d** Relative expression of mRNA for C/EBPβ in astrocytes exposed recombinant HIV proteins compared to vehicle-treated astrocytes. **e** Immunoblot for C/EBPβ using nuclear-enriched lysates from astrocytes exposed to vehicle or recombinant HIV proteins. **f** Quantification of densitometry of the ~ 42 kDa band corresponding to C/EBPβ protein. Statistical analyses were performed by two-way ANOVA followed by Bonferonni’s post-hoc test

To determine if HIV proteins may alter C/EBPβ expression, we treated cultured neuronal and astroglial cells with gp120 (100 ng/ml), nef (100 ng/ml), or Tat (10 ng/ml) for one or four days. Total RNA or nuclear protein lysates were isolated for rt^2^PCR and immunoblot analyses, respectively. HIV Tat, but not gp120 or nef, significantly reduced neuronal C/EBPβ mRNA relative to vehicle-treated cells after one and four days of exposure (*p* < 0.05; Fig. 5a). HIV Tat reduced the band corresponding to C/EBPβ signal in neuronal lysates by 30% compared to vehicle-treated cells after 4 days of treatment (*p* < 0.05; Fig. 5b, c). Conversely, HIV nef and Tat increased astroglial C/EBPβ mRNA expression after 1 day of treatment (*p* < 0.01 and *p* < 0.0001, respectively; Fig. 5d). Tat significantly increased nuclear C/EBPβ expression relative to vehicle-treated astroglia as measured by immunoblot and densitometry analyses (*p* < 0.05; Fig. 5e, f).

## Discussion

In the current study, we present RNAseq and transcriptomic analyses, neuropathological, and biochemical data from FC brain tissues from a well characterized cohort of PWH. These findings support a role for the TF C/EBPβ-mediated alterations in gene expression related to immune response, metabolic pathways, and autophagy in HAND. We report for the first time that C/EBPβ expression is predominantly neuronal in CN brains. However, in HAND brains, C/EBPβ is reduced in neurons and increased in astroglia. We found that C/EBPβ mRNA and protein levels were increased in a cohort of HAND brains compared to brains from CN HIV+ cases. Additionally, transcriptomic analyses confirmed increased C/EBPβ mRNA and uncovered astrocyte marker genes among the differentially regulated C/EBPβ targets. The role of C/EBPβ in astroglia was further investigated by overexpressing C/EBPβ in in vitro models for human astroglia. Multiple genes from the transcriptome of the HAND brains were overexpressed in the astroglia transfected with pC/EBPβ. These findings are consistent with previous reports that show C/EBPβ activity, and expression is altered in neurodegenerative disorders [6], and provides cell-type specific C/EBPβ expression patterns in the FC from CN and HAND brains. RNAseq offers a wide dynamic detection range and does not suffer from hybridization-based limitation associated with microarray such as background noise and saturation, or with probe set issues such as incorrect annotation and isoform coverage. Our findings are consistent with previous reports using gene arrays in which mitochondrial function and inflammation were found to be altered [43, 44]. Some of the highly upregulated genes including TRIM69, CTSB, B2M, UBE2L6, HLA, and BTB3A3 have been previously reported to be associated with HAND [40]. The findings presented here also support an overlap in neuropathogenic mechanisms between HAND and AD (**Table S4**), as has recently been the subject of multiple investigations [45–48]. The current study extends these findings by identifying transcriptional deregulation in MND, specifically C/EBPβ and linking its targets to marker genes in astroglia. This may be particularly important as astroglia have been recently implicated in metabolic complications of HAND and AD [49–54]. Also, consistent with the findings presented here, a recent study showed that YKL-40, a biomarker that reflects astroglial activation, is upregulated in cerebrospinal fluid from HAD cases and is associated with axonal injury [39]. These studies also illustrate a promising strategy to identify cell-specific alterations in brains.

The transcriptome of HIV+ brains has been investigated in previous studies using gene arrays [43, 44]. However, to our knowledge, this is the first time the transcriptome has been investigated in HIV+ brains by traditional neuropathology and biochemical methods with novel RNAseq and systems biology approaches.

The altered transcripts indicating innate immune responses via the toll-like receptors, Fc gamma receptors, TF NF-κB, cytokine type I interferons, and MHC class I molecules are consistent with astrocyte response when exposed to HIV-1 [55–57]. Upregulation of ILF3 and IRAK1, key genes in innate antiviral immune response along with DKK3, an antagonist of Wnt signaling, supports the theory that cytokines prime astrocytes thereby aiding productive viral replication [58]. Alterations in glutamate uptake and potassium channels are associated with HIV-1 infection [59]. Increased expression of calcium-binding receptors (CALM1, CALM2) could be the result of excessive glutamate which can trigger increased levels of intracellular calcium in astrocytes. Nitrosative stress through increased nitric oxide production, known to be triggered by HIV protein Tat, could mediate mitochondrial dysfunction in HIV-mediated neuropathology [60, 61]. Our current data are also consistent with previous reports that show C/EBPβ is upregulated in immune-activated astroglia in vitro, in animal models of Alzheimer’s and Huntington’s diseases and in the brains of donors with neurodegenerative disease [8, 11, 12, 62]. Taken together with previous reports, our current results suggest that C/EBPβ is active in neurons in healthy brains, but neurons downregulate C/EBPβ in the context of HIV infection of the brain, while astrocytes increase C/EBPβ expression in HAND brains. The observation of an overall net increase in C/EBPβ protein levels in the brain despite the reduction in neuronal expression may further suggest that astroglia, and maybe other brain cells, robustly increase C/EBPβ expression. This may reflect a sustained attempt by the host to mitigate neuronal loss and rid the brain of infection through astroglial immune responses. This is supported by reports that C/EBPβ controls transcription of genes regulating neurogenesis and responses to axonal injury in neurons and inflammation in astroglia [6, 7, 11, 12, 63]. ART drugs reduce neurogenesis in the brains of mice [64], which may be consistent with reduced C/EBPβ in neurons. However, on average, the MND cases spent fewer months on ART than CN, suggesting that HIV or inflammation may cause reduced C/EBPβ in neurons. Our finding that Tat reduces C/EBPβ in neuronal cells may partially explain the reductions observed in HIV+ human tissues. This activity of Tat is important because there is strong evidence that Tat is expressed in the brains of PWH [65]. Further investigations of C/EBPβ levels and localization in animal models expressing Tat in the brain and also mechanistic studies using in vitro models are needed to fully understand how neuronal C/EBPβ is reduced in PWH on ART. It is also important to understand how HIV infection of brain cells is related to C/EBPβ expression and localization. Astrocytes harbor HIV-DNA and possibly express HIV proteins [66, 67]. However, recent studies suggest that HIV infection of astrocytes is reduced or non-existent in brains of PWH on ART [68]. Nevertheless, the findings presented here are consistent with our recent report showing that tenofovir disproxil fumarate activates astroglia and increases inflammatory cytokines [69]. Moreover, HIV and inflammatory cytokines increase C/EBPβ expression by astrocytes [11]. Future studies and novel techniques are needed to determine if HIV infection correlates with C/EBPβ expression in astrocytes. Such knowledge could lead to therapeutic targeting of C/EBPβ transcription activity that could potentially modulate inflammation while restoring neuronal activity.

C/EBPβ is a TF involved in immune cell development, inflammatory responses, transcription from the HIV promoter, axonal injury, neurogenesis, and autophagy regulation [7, 11, 12, 70, 71]. The in vitro findings in this report support a role for C/EBPβ in autophagy and mitochondrial function in astroglia during HAND. Moreover, these findings corroborate several studies that have implicated C/EBPβ in regulating autophagy [70, 72]. Although, overexpressing C/EBPβ in astroglia may reveal some specific transcriptional activity of the TF, in an inflamed brain many other TFs are regulated (**Table S5**) and working in concert with C/EBPβ to affect astroglial gene expression. This may explain why overexpressing C/EBPβ had no effect on PINK1 and only marginal effect on BCL11b transcript levels in astroglia. Studies using mouse models for HIV-induced neurotoxicity or other neurodegenerative diseases may offer a platform to better understand how C/EBPβ contributes to neuropathogenesis.

The findings presented here should be viewed in the context of certain limitations of the current study. This study is limited by the fact that the findings in postmortem human brain tissues are largely associative and defining the mechanisms underlying reduced neuronal C/EBPβ in all cases cannot be definitively determined from these studies. Moreover, the mechanisms may vary between cases based on genetic and environmental factors. In vitro studies using neuronal and astrocyte models from different genetic backgrounds may be helpful in determining if HIV proteins, ART, inflammation, or other stimuli may cause alterations in C/EBPβ. However, in vitro studies of dividing astrocytes and neuronal cells cannot fully replicate the disease setting; in vivo studies may provide further insight to the mechanisms underlying shifts in C/EBPβ expression in different brain cell types. It is also noteworthy that while the postmortem tissues analyzed here were from cases that were exposed to ART, regimens change rapidly as newer drugs have come to the market. Also, many of these cases may have lived with HIV before the wide-spread implementation of combination ART, which is not the situation for many people currently living with HIV, and the findings must be viewed in this context. While all the cases were exposed to ART, not all were on ART during the final neurocognitive assessment, which was within one year of death, which may confound the generalization of the mechanisms causing altered C/EBPβ levels. Due to intermittent lapses in records of ART regimens, presumably due to absences from clinical appointments, the continuous duration of ART is not known for all cases. Therefore, effects of ART on C/EBPβ cannot be determined from analyses of the postmortem specimens. All available clinical data for ART regimens for these cases are available through the NNTC database after an embargo. The RNAseq data for each case presented here will also be available in the NNTC database. Importantly, other brain cells, mainly microglia, are involved in neuroimmune signaling in the brain, and this study only focuses on the expression of C/EBPβ in neurons and astroglia. Investigation of C/EBPβ levels in other cell types in the brain, microglia and oligodendroglia, for example, is needed to better understand the overall changes in C/EBPβ protein expression in the brain.

## Conclusion

These findings support a role for C/EBPβ dysregulation as a pathogenic mechanism underlying HAND. These data also suggest that cell-specific targeting of TFs may be used to modulate neuronal and glial function. Future studies focusing on restoring neuronal C/EBPβ and modulating astroglial C/EBPβ function may facilitate proper neuronal function and reduce neuroinflammation in HAND patients. Investigations of C/EBPβ in neurodegenerative disorders should focus on cell-type-specific pathways to design therapeutics targeting this prolific TF.

## Supplementary information

**Additional file 1: Table S1.** Related to Figure 2. Full differential gene expression (DEG) between Minor Neurocognitive Disorder and HIV+ cognitively normal subjects (CNHIV+). **Table S2.** Related to Figure 2. Enriched cellular components between Minor Neurocognitive Disorder and HIV+ cognitively normal subjects (CNHIV+). **Table S3.** Related to Figure 2. Enriched biological processes between Minor Neurocognitive Disorder and HIV+ cognitively normal subjects (CNHIV+). **Table S4.** Disease-gene associations for differentially regulated genes in Minor Neurocognitive Disorders (MND) compared to HIV+ cognitively normal subjects (CNHIV+). **Table S5.** List of differentially regulated transcription factors and along with targets also differentially expressed in Minor Neurocognitive Disorder compared to HIV+ cognitively normal subjects (CNHIV+). **Table S6.** Related to Figure 2. All enriched pathways for C/EBPβ targets in Minor Neurocognitive Disorder compared to HIV+ cognitively normal subjects (CNHIV+). **Table S7.** Related to Figure 3. List of astrocyte specific marker genes that are also C/EBPβ targets in Minor Neurocognitive Disorder compared to HIV+ cognitively normal subjects (CNHIV+). **Table S8.** Related to Figure 3. All enriched pathways for C/EBPβ regulated astrocyte marker genes targets in Minor Neurocognitive Disorder compared to HIV+ cognitively normal subjects (CNHIV+).

**Additional file 2: Figure S1.** KEGG pathways shows distinct mechanisms between the C/EBPβ up and downregulated gene sets. Bar plots show the distinct pathways between the upregulated and down regulated target genes of C/EBPβ. The pathways are sorted by p-value which is calculated using the Fischer’s exact test.

## Data Availability

All data and materials will be provided as available upon request. Data generated from postmortem human samples will be deposited in the National NeuroAIDS Tissue Consortium database.

## Abbreviations

HAND: HIV-associated neurocognitive disorder
FC: Frontal cortex
C/EBP: CCAAT enhancer binding protein
TF: Transcription Factor
MND: Minor neurocognitive disorder
CN: Cognitive normal
HIV: Human immunodeficiency virus
PWH: People with HIV
ANI: Asymptomatic neurocognitive Impairment
HAD: HIV-associated dementia
IL: Interleukin
NNTC: National NeuroAIDS Tissue Consortium
VL: Viral Load
PBST: Phosphate-buffered saline-tween 20
PBS: Phosphate buffered saline
P: Plasmid
CNHIV+: HIV+ Cognitive normal subjects
Cpm: Counts per million
Gsea: Gene set enrichment analysis
PMI: Postmortem interval
SLC1A2/3: Subunit of glutamate ionotropic receptor and glutamate transporters
CALM1/2: Calcium binding receptors
pCON: Control plasmid
