# Supplementary material for: Transcriptomic analysis of brain tissues identifies a role for CCAAT enhancer binding protein β in HIV-associated neurocognitive disorder
Source: J Neuroinflammation. 2020 Apr 11;17:112. doi: 10.1186/s12974-020-01781-w (PMC7149918; doi:10.1186/s12974-020-01781-w)

Upregulated Gene set

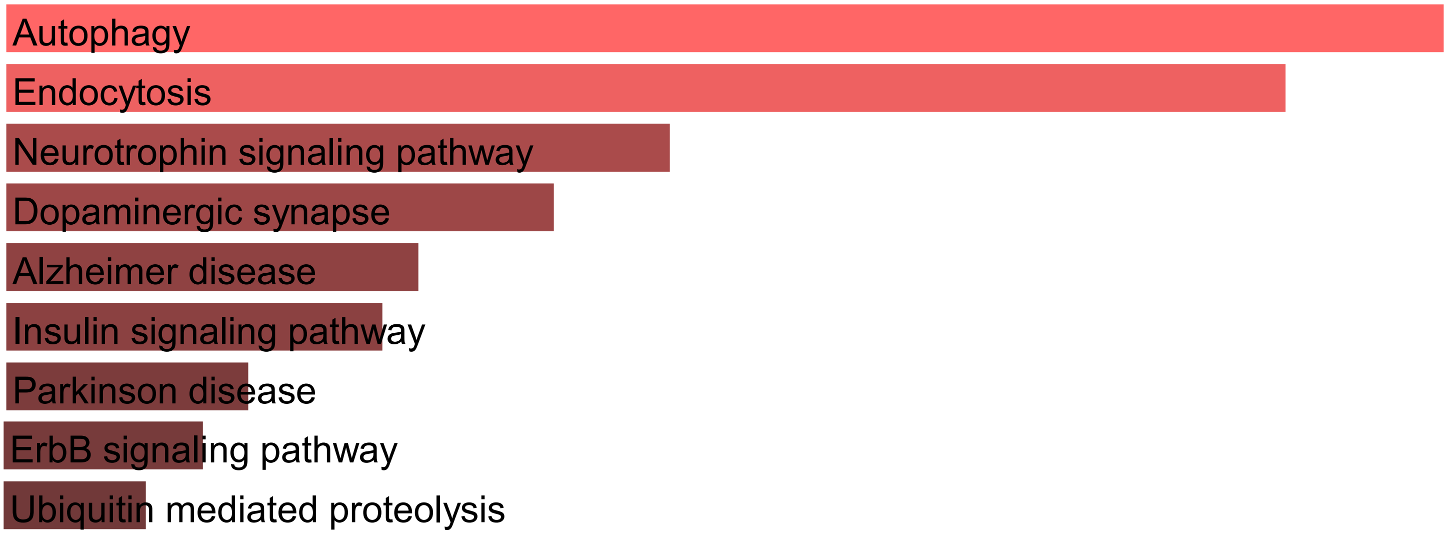

Downregulated Gene set

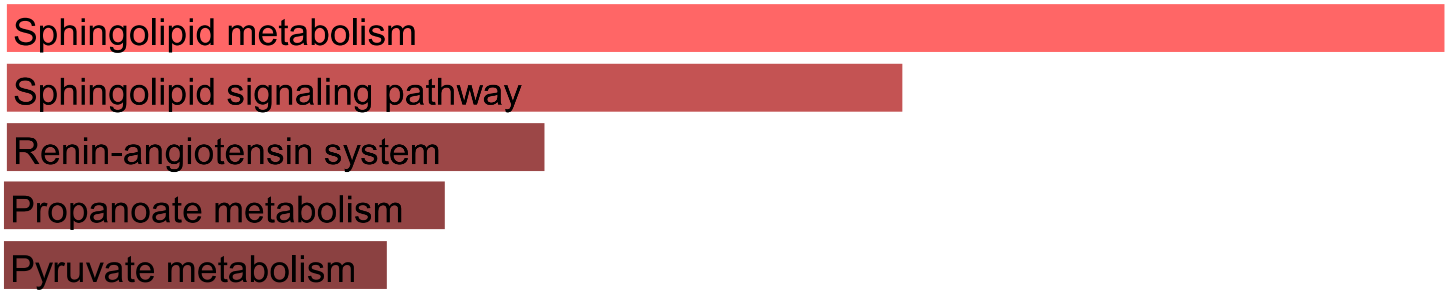

Supplement: Supplementary file 2 — Additional file 2: Figure S1. KEGG pathways shows distinct mechanisms between the C/EBPβ up and downregulated gene sets. Bar plots show the distinct pathways between the upregulated and down regulated target genes of C/EBPβ. The pathways are sorted by p-value which is calculated using the Fischer’s exact test. [file 12974_2020_1781_MOESM2_ESM.pdf]
